# Fracture of the Sacrum Impeding Delivery

**Published:** 1846-04

**Authors:** A. H. David

**Affiliations:** Montreal


*Fracture of the Sacrum impeding Delivery.*—By A. H. David, M.D., Montreal.—Madame C——, of delicate frame, 30 years of age, about four months pregnant with her fourth child, was coming down stairs, when her foot slipping, she fell and bounded on several steps till she reached the bottom one. She immediately felt excruciating pain in the region of the sacrum, and was obliged to be carried to her sofa, as she was quite unable to rise. The pain continuing, her ordinary medical attendant was called to see her. He bled her and gave her an anodyne, and ordered the part to be rubbed with strong camphorated liniment; he was very anxious to make an examination to discover the cause of the great pain, but she would not consent to his doing so. The pain continued for some weeks, but gradually subsided, and some three months after the accident she was able to attend to her ordinary duties for a short time, but the pain soon returned again, and continued with such unabated violence, till the full time of her pregnancy, as to induce serious apprehensions for her safety.

Labour commenced on the 22d, June, and Dr. Badeau finding it impossible to deliver, requested me to see her with him, after she had been about five and twenty hours in labour. The pains were very active, and as far as could be ascertained, the os uteri was fully dilated, but the vagina was almost completely closed by a hard tumour, connected, apparently, with the sacrum, and, on inquiring into the case,we learned the foregoing history. She was exceedingly weak and emaciated, in consequence of the length of time she had been suffering, viz., from the end of January to the end of June; and from the certainty that the child had been dead for some time, I suggested the propriety of immediately performing craniotomy, in which Dr. Badeau fully coincided with me. After much loss of time before Madame C—— could make up her mind to submit to the operation, I proceeded, towards the morning of the 24th, to break down the head, and succeeded with more ease than I had anticipated from the closure of the vagina. After completely succeeding in this, I desisted from proceeding to extract the child for a short time, at her earnest request, and on my wishing to resume some two hours after, she most distinctly refused to allow me to do anything ; and notwithstanding the repeated wishes of her priest, who was with her all the time, and the entreaties of her husband and friends, declared her readiness to die sooner than allow herself to be touched, and as she persisted in this determination, we had no course left but to see her gradually sink before our eyes, and during her expiring efforts the mutilated remains of her child came from her.

Having obtained permission to make an examination of the sacrum, we found that bone broken into four pieces; two of them projected out of its usual line, and all were joined by a firm callous band, which formed the projection into the vagina, and completely prevented a natural discovery.—*Brit. Am. Journ. of Md. and Phys. Sci.*

